# Synthesis and Corrosion Resistance of FeMnNiAlC_10_ Multi-Principal Element Compound

**DOI:** 10.3390/ma14216356

**Published:** 2021-10-24

**Authors:** Mohammed Hussien, Karl Walton, Vladimir Vishnyakov

**Affiliations:** Centre for Engineering Materials, University of Huddersfield, Huddersfield HD1 3DH, UK; k.walton@hud.ac.uk (K.W.); v.vishnyakov@hud.ac.uk (V.V.)

**Keywords:** FeMnNiAlC_10_, multi-principle alloy, high-entropy alloy (HEA), thin film, corrosion resistance, hardness, Young’s modulus

## Abstract

A multi-principal element FeMnNiAlC_10_ bulk alloy was produced by vacuum arc melting. The same alloy was sintered as a thin film on a silicon substrate by ion beam sputter deposition. The bulk alloy has a multiphase structure the elements predominantly segregating into iron manganese carbides and nickel aluminium phases. The thin film is amorphous without detectable phase segregations. The absence of segregation is attributed to the film composition and deposition onto substrate at temperature below 400 K. The corrosion resistance of the thin film alloy was evaluated in 3.5% NaCl. The FeMnNiAlC_10_ thin film alloy has better corrosion resistance than 304SS. The hardness of the thin film was approximately 7.2 ± 0.3 GPa and the reduced Young’s modulus was approximately 103 ± 4.6 GPa. FeMnNiAlC_10_ thin film could be a good candidate for coating oil and gas extraction soft iron infrastructure.

## 1. Introduction

Engineering alloys need to satisfy an ever-increasing list of requirements, and still be economical for the given application. It has been shown recently that by breaking the classical alloy design philosophy and having more elements in high concentrations, rather than having one principle element, it is possibly to enhance alloy properties. It was noted recently that maximising the entropy of mixing can enhance alloy properties. If the elements are mixed in equal or close to equal concentrations alloy performance is superior compared to conventional alloys [1,2,3]. The former type of alloys is known as highly concentrated alloys or high-entropy alloys. Typically, HEAs are defined as alloys which contains five or more elements in equal atomic ratios [4,5,6,7,8]. However, the effect of entropy enhancement is still noticeable even with just three or four elements and with not exactly equal element concentrations. It was shown that positive effects are observed even when elemental concentrations vary within the range of 5 to 35 at% [9,10,11,12,13].

Many studies revealed that highly concentrated alloys have superior properties in numerous applications compared to some conventional alloys [14]. As a result, HEAs attracted extensive scientific and industrial communities’ attention [15,16,17]. These alloys open new paths and applications due to their properties such as: good structural stability, high wear resistance, enhanced corrosion resistance, excellent thermal stability and high hardness [18,19,20,21,22,23]. In some cases, the alloys tend to form single-phase structures with a high symmetry due to their high mixing entropy. These structures encompass face-centred cubic (fcc), body-centred cubic (bcc), and hexagonal close-packed (hcp) [23,24,25,26,27]. Moreover, some HEAs can be amorphous [28,29,30]. At the same time, many alloys with enhanced entropy demonstrate phase separation and are multi-phase alloy. Nevertheless, the alloys have demonstrated significant useful properties [31,32,33]. Phase separation and grain boundaries are known to affect mechanical properties, for instance, the corrosion resistance for the alloy will only be as good as the weakest phase. Furthermore, grain boundaries are typically an unhomogenised weak phase, thus leading to weak points which limit the alloys properties.

In the bulk alloy case during the vacuum arc melting process, there is sufficient time for the material to reach thermodynamic equilibrium and develop phase separation and element segregation due to the relatively slow cooling. However, in the thin film coating process, the thermodynamic state is expected to be far from the equilibrium point. The thin film material might have some immediate benefits compared to the bulk material nevertheless properties can change over time and at elevated temperature. On the positive side, thin film HEAs can be used as coatings. This approach is better than using HEAs as bulk as their beneficial properties can be exploited at low cost. [34]. Consequently, many HEA coatings have been investigated recently, being deposited by various thin film techniques such as laser cladding [35], plasma arc cladding [36], electro spark process [37] and magnetron sputtering [38,39,40].

In oil and gas recovery, the extracted multi-phase fluid usually contains some corrosive electrolytes such as dissolved mineral water solutions and acids such as H_2_S. These solutions readily interact with the surfaces of the production network pipes and storage vessels and in many cases, this leads to corrosion damage. In which case the practical approach is to use alloys with high corrosion resistance such as those containing Cr and/or Ni. Transporting fluids containing sand also abrades the containment structures which necessitates high material hardness and abrasion resistance.

While many papers report on the mechanical properties of HEAs, offering data on hardness, ductility and strength [41,42]., there are not so many studies focusing on corrosion resistance.

Most HEAs are based on traditional metal alloying elements such as Al, Cu, Cr, Fe, Mn, Mg, Ni and Ti [43]. There is evidence that adding metalloids such as Carbon or Boron [44,45], can enhance the corrosion resistance of the alloy. Just a few HEAs with carbon have been investigated so far [46]. In the alloys that have been studied, such as Al_0.3_CoCrFeNiC_0.1_ and Fe_40.4_Ni_11.3_Mn_34.8_Al_7.5_Cr_6_ the addition of 1.1 at% carbon, enhances mechanical properties [47]. Adding carbon to CoCrFeNiC_*x*_HEAs increases the strength, hardness and wear resistance [48].

The aim of this study was to produce HEA material with good protection properties which include high corrosion resistance and elevated hardness. Both bulk and HEA thin film (HEATF) alloys of FeMnNiAlC_10_ (C_10_ marks alloy with 10 at% carbon) highly concentrated alloy have been produced. Given protection of conventional material is done by thin film coating, the focus in this study is thin film properties. In this context, sintered bulk alloy was used to check material thermodynamic stability towards phase segregation.

## 2. Experimental Methods

The bulk alloy was produced by vacuum arc melting from a pure powder mixture of Fe, Mn, Ni, Al, and C. The purity of the element powders was approximately 99.9%. The melt process was carried out under a continuous flow of protective argon at approximately 0.5 atm. As in many studies of HEAs [39,49,50,51]. the re-melting process was repeated three times to guarantee chemical homogeneity.

The thin film alloy was generated by ion beam sputter deposition. Separate elemental targets were used and located under 4” sputtering beams to achieve the desired thin film composition. Argon was used as a working gas. The films were deposited on silicon (1,1,1) substrate (silicon wafer with native oxide and 400 micrometres overall thickness). After two hours of deposition the deposited film thickness was approximately 1.2 ± 0.1 micrometres as measured by cross-sectional imaging in SEM.

X-Ray Diffraction (XRD) was used to observe the material structure. The chemical composition was analysed with Energy Dispersive X-Ray (EDX) analysis with ZAF correction in a Field Emission Gun Scanning Electron Microscope (FEG SEM).

The material corrosion behaviour was tested by potentiodynamic polarisation measurements. A cell with three electrodes was used with saturated calomel reference electrode and a platinum counter electrode. The measurements were done in 3.5% NaCl aerated by nitrogen at room temperature and under atmosphere pressure.

The material was polarised anodically with a scanning rate at close to 0.017 V/s as required by ASTM International standard (American Society for Testing and Materials) [52]. For corrosion behaviour comparison a SS 304 sample was used. All materials were tested with the same standard parameters. The samples were put in the solution and kept for 600 s to reach Open Circuit Potential (OCP) stability. After that the materials were scanned anodically between −0.1 to 1.5 V versus OCP with a 0.02 V/s scanning rate.

A nanoindentation system (Micro Materials Ltd., Wrexham, UK) was used to measure the mechanical properties. The hardness was determined using Micro Materials software with Power Law Fitting between 100 and 20% of maximum load and then using Oliver-Pharr formalism.

## 3. Results and Discussion

**Figure 1:**
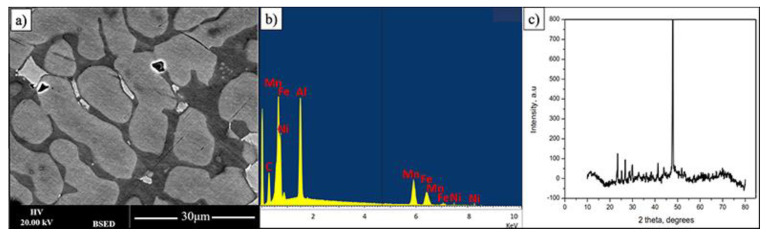
(**a**,**b**) BSE image and its EDX spectrum displaying three phases of FeMnNiAlC_10_ bulk alloy processed by arc melting, (**c**) is the alloy XRD pattern.

As the intensity of Backscattered Electrons is governed by an averaged atomic weight this makes it possible for fast phase presence observation. Figure 1 shows the BSED (backscatter electron detector) SEM image of FeMnNiAlC_10_ bulk alloy surface after polishing. The overall alloy has FeMnNiAlC_10_ (where C_10_ signifies 10 at% of carbon) composition (see Table 1). While the overall alloy has almost equiatomic metal composition it is possible to see that there are at least three phases with two predominant ones. For simplicity of description, they will be named bright, grey and dark. The overall sample X-Ray diffraction pattern demonstrates one high-intensity peak at 47.15° and a series of peaks of low-intensity at lower angles. Given multiphase material nature it is impossible at this point to provide XRD phase identification.

The material composition and presented phases are summarised in Table 1.

**Figure 2:**
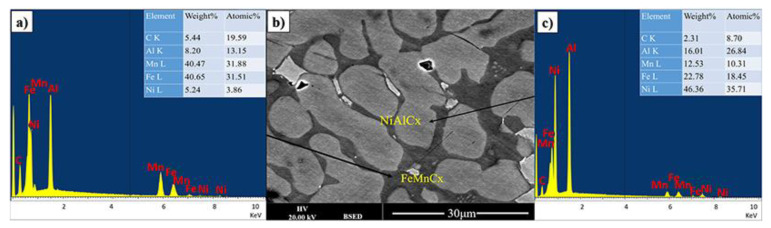
(**b**) BSE image, (**a**,**c**) are EDX spectrum from two main phases of FeMnNiAlC_10_ bulk alloy processed by arc melting.

The dark appearing phase is predominantly composed of iron, manganese and carbon with significant amount of Al (see Figure 2). The brighter appearing phases contain less carbon and iron. From this it can be tentatively concluded that the carbon in the material mostly interacts with iron. However, in this case, it is also presumed that it is not standard cementite as all iron cementites have most intense XRD peaks below 46^0^ (JCPDS 36-0772, JCPDS 36-1248, JCPDS 36-1249), which are only observe to a small extend. It can only be further speculated that the cementite structure is further modified by the presence of Mn and Al.

In the bright appearing phase there is a dominance of Ni and Al with some iron and significantly reduced Mn. It is possible to refer to a predominantly NiAlFe phase for both grey and bright appearing crystallites.

It is stressed that analysis of the bulk material was only done to determine if the material will phase separate at elevated temperatures. The main goal was to investigate the properties of the composition in thin film form as a protective coating.

**Figure 3:**
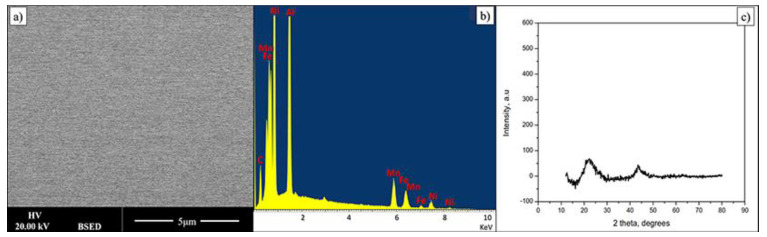
(**a**,**b**) BSE image and its EDX spectrum displaying a single phase of FeMnNiAlC_10_ HEATF, (**c**) is the film XRD pattern.

Thin film composition is presented in Table 2. Within quantification error the material is very similar in chemical composition to the bulk alloy. Figure 3 shows data related to FeMnNiAlC_10_ HEA thin film. The electron backscattering reveals a compositionally uniform single-phase material. In addition, the X-Ray diffraction pattern of the AlFeMnNiC_10_ thin film shows two low-intensity peaks, indicating the film is amorphous. During deposition the Si substrate is not intentionally heated; however it has been noted that under these deposition conditions it does reach 400 k. In this particular case this temperature is not high enough for film crystallisation to occur or to lead to phase separation. Data on bulk alloys of similar composition show that the film may crystallise and the phases will probably separate but the activation temperature for this is currently not known though it will be determined in future experiments.

## 4. Potentiodynamic Polarisation

**Figure 4:**
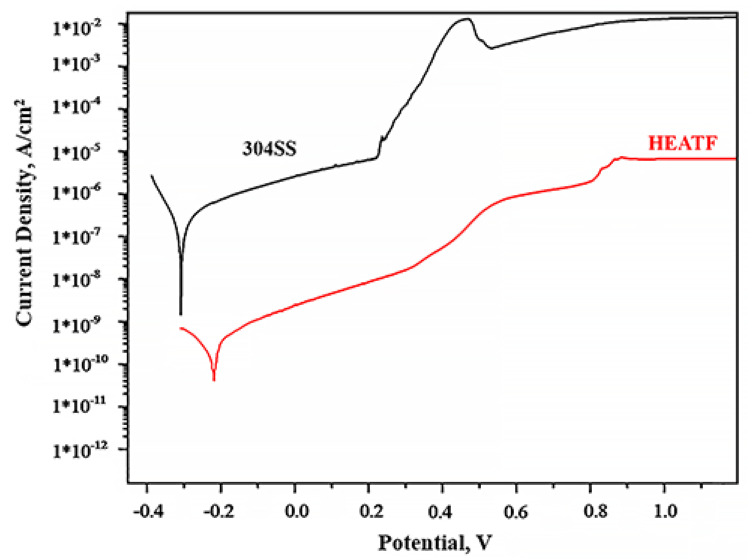
Potentiodynamic polarisation curves for 304SS and FeMnNiAlC_10_ HEATF.

Figure 4 shows the potentiodynamic polarisation curve results for FeMnNiAlC_10_ thin film and 304 Stainless steel. The corrosion parameters such as corrosion current density and corrosion potential of FeMnNiAlC_10_ and 304 SS are presented in Table 3.

The corrosion resistance electrochemical analysis theory states that a material is resistant to corrosion when it shows small corrosion current density and elevated corrosion potential.

It is possible to see that the thin film is more corrosion resistant than 304SS (−0.21 V against the −0.3 V) and at the comparable potential the thin film has more than two order of magnitude lower corrosion current density. The pitting potential for 304SS is observed at 0.25 V, while gradual increase of corrosion current in the thin film case is only observed after 0.37 V, even then the corrosion current does not increase abruptly until 0.82 V. This slow increase in corrosion current, apart from chemical composition is most likely due to the absence of grain boundaries in the amorphous thin film. It is well known that corrosion in alloys begins with chemical reactions on grain boundaries which are absent in the case of amorphous material. Moreover, additional alloy corrosion resistance enhancement is believed to be due to adding metalloids such as Carbon [44]. All the above indicate that FeMnNiAlC_10_ thin film is superior to 304SS and will be an effective corrosion protection coating.

The results also demonstrate the HEATF much lower corrosion current as compared to some previously published work. For instance, 1.5*10^−10^ A/cm^2^ for FeMnNiAlC_10_ thin film against 1.315 × 10^−5^ A/cm^2^ for AlCrFe_2_Ni_2_W_0.2_Mo_0.75_ [53]. Density of corrosion current is above two orders of magnitude lower than reported by Nene et al. [54]. The high corrosion resistance is probably attributed to a single phase and high concentration of carbon (or carbide, in this case).

## 5. Mechanical Properties

**Figure 5:**
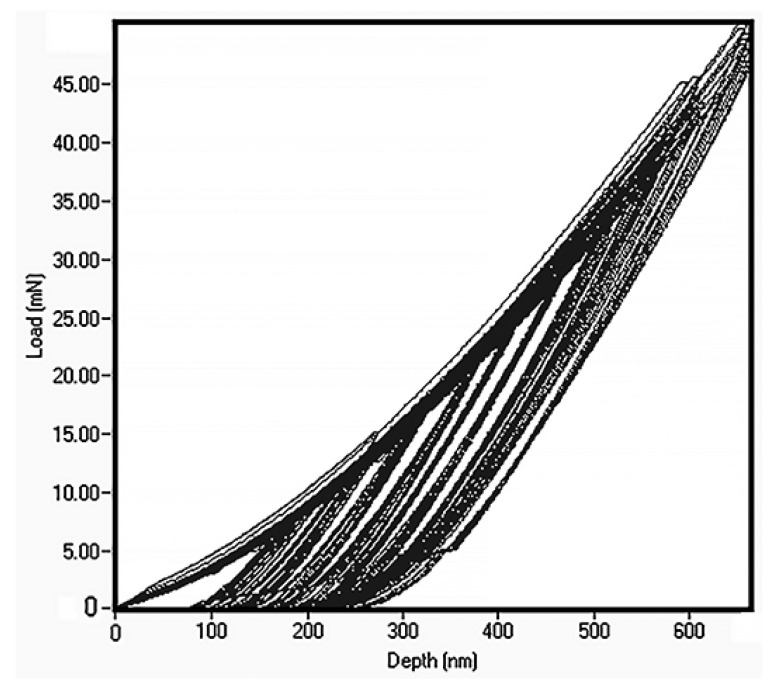
Nanoindentation data for FeMnNiAlC_10_ amorphous HEATF.

The thin film alloy mechanical parameters were measured by nanoindentation (see Figure 5). The load was gradually increased from 10 mN and to 50 mN. The data show that this led to Berkovich indenter penetration depth to ^1^/_2_ of the film thickness. In many cases, this could be regarded as constituting excessive penetration, as ^1^/_10_ of thin film thickness is a more typical penetration for determination of thin film properties. However, in this case, it is important to keep in mind that the hardness of the silicon, which is the substrate, is 11 GPa [31]. Within the loading range the hardness demonstrated absence of penetration depth dependence and we calculated hardness as an average value, which leads to the 7.2 ± 0.3 GPa.

**Figure 6:**
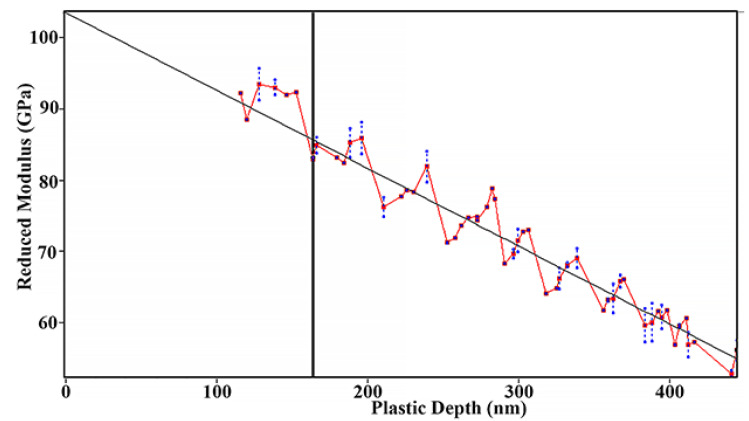
Reduced Young’s modulus of amorphous FeMnNiAlC_10_ thin film.

The true value of the reduced Young’s modulus should be determined at zero load as prescribed by the international standard (ISO 14577-4:2016). Linear extrapolation to zero penetration depth gives 103 ± 4.6 GPa for the thin film as evident from Figure 6.

The material property values obtained can be analysed in terms of the material’s composition-structure. The obtained hardness is relatively high for a predominantly metallic film but not so high as ceramics. In many cases, hardness of high-speed steels, M4 for example, is just above 4 GPa and only in the case of nickel superalloys the hardness reaches area at around 9 GPa. As for ceramics, then the hardness reaches 30–40 GPa for many engineering nitrides or carbides [55,56]. As hardness is partially related to a presence of ceramic-like structures we can relate the material hardness to the presence of carbides in the material. However, iron carbide (which does not exist as a separate phase in the amorphous film, but iron carbon bonds will exist) by itself in crystalline form only has hardness at just above 10 GPa and a Young modulus below 200 GPa [57]. The presence of manganese shifts the alloy properties toward a material which is well known as Hadfield steel or mangalloy, valued for high impact strengths and resistance to abrasion in the mining industry. Beneficial contributions can also be expected from the random mixture of the constituent atoms and the presence of carbon.

## 6. Conclusions

High concentrated alloy FeMnNiAlC_10_ was prepared by two production routes. The bulk alloy was synthesised by vacuum arc melting and the thin film was synthesised by ion beam sputter deposition. The bulk alloy shows phase separation, which indicates possible thermodynamic instability at elevated temperatures. The material splits into two distinctive phases. One predominantly contains carbon with iron and manganese and another has high concentrations of nickel and aluminium (approximate compositions can be written as Fe_8_Mn_8_NiAl_3_C_5_ and Fe_7_Mn_4_Ni_10_Al_9_C_3_). The thin film was uniform and amorphous without any phase segregations. The FeMnNiAlC_10_ thin film has higher corrosion resistance than 304SS in aqueous 3.5% NaCl solution under atmospheric pressure and at room temperature. Furthermore, the HEATF alloy’s hardness was found to be 7.2 ± 0.3 GPa with a reduced Young’s modulus at approximately 103 ± 4.6 GPa.

Hence, it is believed that the FeMnNiAlC_10_ amorphous thin film is suitable for use as protection for mild steel in severe environments, such as the processing and exploration of oil and gas where high corrosion resistance should be supported by high resilience to mineral abrasion.

## Figures and Tables

**Table 1:** Chemical composition of materials and present phases.

Area of Analysis	Element Atomic%, ±4% Error					
	C	Al	Mn	Fe	Ni	Total
Overall composition	11.9	22.0	17.3	23.2	25.6	100
Dark phase	19.6	13.2	31.9	31.5	3.9	100
Grey phase	8.7	26.8	10.3	18.5	35.7	100
Bright phase	8.7	26.0	12.1	20.8	32.4	100

**Table 2:** The chemical compositions for bulk and thin films alloys.

Alloy Type	Element Atomic%, ±4% Error					
	C	Al	Mn	Fe	Ni	Total
Bulk Alloy	11.9	22.0	17.3	23.22	25.6	100
Thin Film	14.6	17.5	20.0	19.7	28.2	100

**Table 3:** Corrosion parameters obtained from potentiodynamic polarisation.

Sample	*I_corr_,* A/cm^2^	*E_corr_,* V	*E_pit_,* V
FeMnNiAlC_10_	1.5*10^-10^	−0.21	0.82
304 SS	10^-7^	−0.30	0.25

## Data Availability

Not applicable.
